# Evidence for Gender-Dependent Genotype by Environment Interaction in Adult Depression

**DOI:** 10.1007/s10519-015-9752-4

**Published:** 2015-10-14

**Authors:** Dylan Molenaar, Christel M. Middeldorp, Gonneke Willemsen, Lannie Ligthart, Michel G. Nivard, Dorret I. Boomsma

**Affiliations:** Psychological Methods, Department of Psychology, University of Amsterdam, Weesperplein 4, 1018 XA Amsterdam, The Netherlands; Department of Biological Psychology, VU University Amsterdam, Amsterdam, The Netherlands; Neuroscience Campus Amsterdam (NCA), Amsterdam, The Netherlands; EMGO+ Institute for Health and Care Research, VU University Medical Center, Amsterdam, The Netherlands

**Keywords:** Adult depression, Common environment, Genotype-by-environment interaction, Heritability, Heterogeneity

## Abstract

Depression in adults is heritable with about 40 % of the phenotypic variance due to additive genetic effects and the remaining phenotypic variance due to unique (unshared) environmental effects. Common environmental effects shared by family members are rarely found in adults. One possible explanation for this finding is that there is an interaction between genes and the environment which may mask effects of the common environment. To test this hypothesis, we investigated genotype by environment interaction in a large sample of female and male adult twins aged 18–70 years. The anxious depression subscale of the Adult Self Report from the Achenbach System of Empirically Based Assessment (Achenbach and Rescorla in Manual for the ASEBA adult: forms and profiles, 2003) was completed by 13,022 twins who participate in longitudinal studies of the Netherlands Twin Register. In a single group analysis, we found genotype by unique environment interaction, but no genotype by common environment interaction. However, when conditioning on gender, we observed genotype by common environment interaction in men, with larger common environmental variance in men who are genetically less at risk to develop depression. Although the effect size of the interaction is characterized by large uncertainty, the results show that there is at least some variance due to the common environment in adult depression in men.

The heritability of depression in adults is estimated at around 40 % (Sullivan et al. 2000; Nivard et al. 2015). Interestingly, in adults the remaining phenotypic variance is consistently found to be solely due to the unique environment. In adolescents, however, at age 12 years variation in anxious depression is explained also by shared environmental factors, while at ages 14 and 16 these shared environmental effects were absent (Lamb et al. 2010). The absence of evidence for common environmental influences on depression after age 12 is remarkable, as it has been argued that, theoretically, at least some phenotypic variance in depression is expected to be due to the familial effects in childhood that persist into adulthood (Gatz et al. 1992). For instance, cognitive styles related to depression may be learned in the family (Monroe and Simons 1991; Mezulis et al. 2006; Ingram 2003), and familial traumatic events in childhood, such as divorce, affect children similarly (Bowlby 1977; Kessler et al. 1997; Silberg et al. 2001). Therefore, it has been argued that the recurring finding of no common environmental effects on adult depression may be spurious.

Duncan et al. (2014) hypothesized that the true effects of the common environment underlying depression are masked by non-linear effects. Specifically, the effects of the common environment (C) may depend on the genotype of the subject (A) that is an A×C interaction. If not explicitly modeled, such an interaction effect is included in the estimate of the genetic variance (Molenaar et al. 1990). The question arises whether common environmental effects on adult depression can be revealed by taking into account such non-linear effects.

Another question is why some individuals develop a depression after an adverse environmental event and others do not. Both linear and non-linear effects could explain this phenomenon. Given the ongoing debate of the usefulness of genetic variant by environment interaction studies, either in a candidate gene study or in a genome-wide association study, it is important to know whether non-linear effects are present for unique environmental effects (see e.g., Dick et al. 2015).

Therefore we investigated whether common and unique environmental variance influencing the vulnerability for adult depression can be detected by taking into account the non-linear effects of genotype by environment interaction. We also tested whether interaction effects differ between males and females. Gender differences in the prevalence of depression arise in adolescence and remain until older age (Kessler et al. 1993). The exact mechanism underlying the higher prevalence of depression in adult females is generally unknown (Piccinelli and Wilkinson 2000), although studies have indicated that environmental factors associated with depression are different for males than for females (Kendler et al. 2011; Klose and Jacobi 2004).

We analyzed data from a large sample of twins between the ages of 18 and 70 years from the Adult Netherlands Twin Register (Nivard et al. 2015). The twins completed the anxious depression subscale of the Adult Self-Report from the Achenbach System of Empirically Based Assessment (Achenbach and Rescorla 2003). We tested for unmeasured genotype by unmeasured environment interaction effects in these data using the heteroscedastic ACE model (Jinks and Fulker 1970; Molenaar et al. 2012). Using the item level data methodology (see Molenaar and Dolan 2014; Schwabe and van den Berg 2014), we modeled a latent depression factor as a function of additive genetic (A), common environment (C), unique environment (E), and non-linear effects (A×E and A×C). In addition, we extended the approach to enable tests on gender differences in these interaction effects. In the resulting model adopted here both the genetic and the environment effects are treated as latent factors. By studying the interaction at the level of the latent genetic and environmental variance, we did not require measured candidate genes and measured candidate environments. In addition, the modeling approach is insensitive to the scale properties of the data, which may otherwise result in spurious non-additive effects (Eaves et al. 1977; Molenaar and Dolan 2014; Schwabe and Van den Berg 2014).

## Method

### Participants and measures

The Netherlands Twin Register (NTR; see Nivard et al. 2015) includes the Young NTR (YNTR; van Beijsterveldt et al. 2013) and the Adult NTR (ANTR; Willemsen et al. 2013). In the YNTR, twins have been registered at birth by their parents since 1987 (Bartels et al. 2007). When twins reach the age of 18, they are enrolled in the ANTR. The ANTR originally included adolescent and adult twins who were recruited through city councils or who volunteered through the NTR website. Here we analyze the data from all twins aged 18 years and older. The dataset comprises 6511 twin pairs (no missing: 5923) between the age of 18 and 70 with information on depression and zygosity. These pairs consist of 3146 (no missing: 2895) are MZ twins and 3365 (no missing: 3028) are DZ twins.

The twin pairs completed the anxious depression subscale of the Adult Self Report (ASR), which is part of the Achenbach System of Empirically Based Assessment (Achenbach and Rescorla 2003). The twins were asked to indicate to what degree various statements concerning anxious depressive behavior and attitudes apply to them on a 3-point scale (‘not true’, somewhat or sometimes true’, ‘very or often true’). The ASR anxious depression subscale was included in 8 of the 11 surveys that have been collected for the NTR since 1991 (respectively in 1991, 1995, 1997, 2000, 2002, 2009, 2011, and 2013). The different surveys contained slightly different versions of the ASR, as over the years, the ASR has changed. However, for anxious depression there was a common set of 13 items included in all surveys that was analyzed in this project (see Appendix 1). Cronbach’s alpha for these items on the various measurement occasions and twin samples ranged between 0.83 and 0.90 which is an indication for good reliability. In addition, the correlations between the sum scores based on these 13 items and the sum scores based on all items at each measurement occasion are between 0.95 and 0.98. The validity of the ASR has been established by Achenbach and Rescorla (2003).

Not all twins participated at each measurement occasion (see Table 1). For instance, 2131 twin-1 members have data available at only one occasion and 1273 twin-2 members have data available at three occasions. We selected the data vector from the first occasion that has the least missing values for each twin. This data vector contains missing values due to twins not completing all items of the questionnaire. Here, we assumed that these missing values are missing at random so that we can take all missing values in the data into account in the model fitting approach described below.

**Table 1:** The number of twin pairs that have data available on none, 1, 2, …, or all measurement occasions

	None	1	2	3	4	5	6	7	All
Twin 1	382	2131	1675	1334	491	444	242	158	41
Twin 2	374	2184	1642	1273	526	451	262	144	42

‘None’ means that only the co-twin has data available on 1 or more measurement occasions

### Analysis

Our main objective is to test for genotype by environment interaction for males and females in the complete sample of twin pairs. However, we first needed to establish that the data are homogeneous with respect to measurement occasion and age. Below we discuss a standard measurement model and the biometric model. Next, within these models, we discuss how we assessed homogeneity of the data with respect to measurement occasion and age. Then, we introduce the gender dependent genotype by environment interactions in the biometric model.

### Standard measurement and biometric model

As advocated by Van den Berg et al. (2007), we analyzed the data at item level by separating between a measurement model for the items and a biometric model for the genetic and environmental variance. In the present study, the so-called graded response measurement model for ordinal item responses was used (Samejima 1969). Using this model, we separated the measurement properties of the item scores, *X*_*i*_, from the underlying latent phenotypic factor (anxious-depression), denoted θ. Here, *p* = 1, *…*, *N* is used to index the twin pairs, *j* = 1, 2 is used to index the twin members, and *i* = 1, …, *n* is used to index the items.

In the graded response measurement model, the observed item scores, *X*_*i*_, are regressed on the latent phenotypic factor, θ (using a multinomial probit regression function). The intercept and slope parameters in this regression are respectively referred to by *threshold* and *discrimination* parameters. These parameters are purported to capture the measurement characteristics of the item scores. In the present case, where we have a three point scale, we have 2 threshold parameters, τ_i1_, and τ_i2_. These parameters model the relative attractiveness of the answer options, that is, the degree to which the subjects use the different answer options. In an extreme depression item, for instance “I often think of suicide”, τ_i1_ will likely be large, reflecting that the first answer option is attractive (most subjects score in the first answer category indicating that they do not think of suicide) and the second option is not. As the item scores are assumed to be ordered, the thresholds are also ordered, that is, τ_i2_ should always be larger than τ_i1_.

**Fig. 1:**
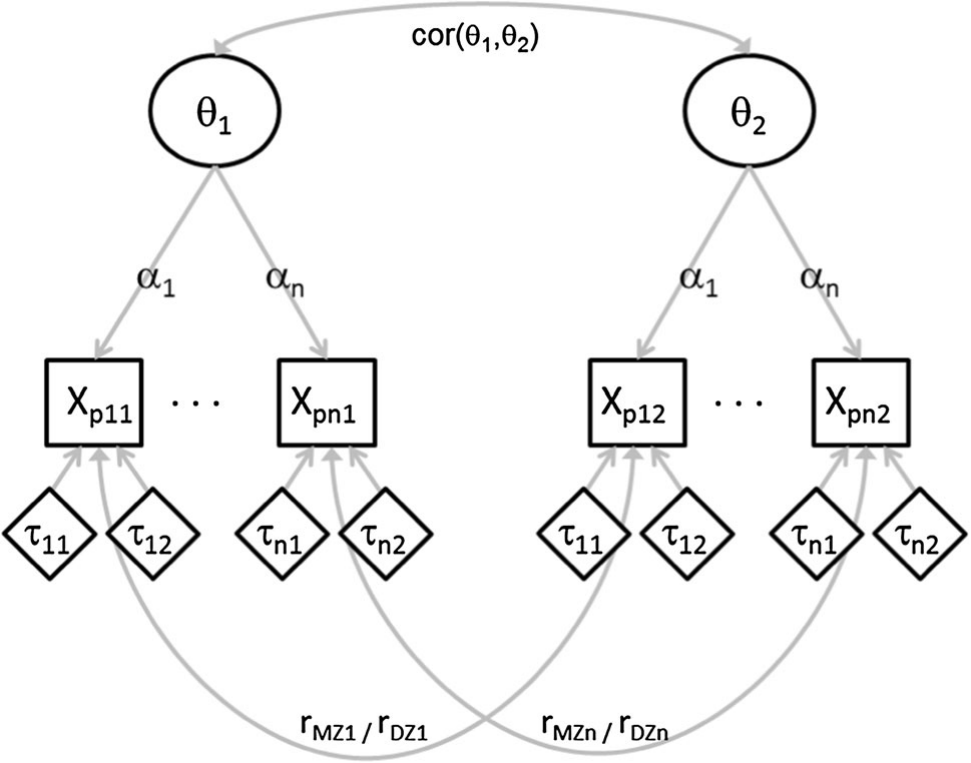
Graphical representation of the measurement model including the parameters

The slope, or discrimination parameter α_i_, in the regression of the item scores on the latent phenotypic variable, models the degree to which the item scores can distinguish between subjects with different levels of θ. The higher the value of α_i_, the better indicator the item is for θ. Besides the threshold and discrimination parameters, measurement models for twin data often also include the *residual correlations* between the item scores of the twin 1 and twin 2 members in the MZ sample (r_MZ,i_) and the DZ sample(r_DZ,i_). Such a correlation may indicate shared item specific genetic and/or environmental variance or it may indicate measurement problems resulting from filling in the questionnaire by two twins together. See Fig. 1 for a graphical representation of the measurement model including the parameters, and see Appendix 2 for a more technical discussion of the measurement model.

As the measurement model above captures the measurement properties of the item data in the α_i_, τ_i1_, τ_i2_, r_MZi_, and r_DZi_ parameters, the latent phenotypic factor, θ, is unaffected by the scale properties in principle.^1^ In the standard phenotypic model, the phenotypic variable, θ, is decomposed into an additive genetic (A), common environmental (C), and unique environmental (E) variance component and an intercept, ν, that is

$$\uptheta_{\text{pj}} = \nu + {\text{A}}_{\text{pj}} + {\text{C}}_{\text{pj}} + {\text{E}}_{\text{pj}}$$

where COR(A_1_, A_2_) = 1 for MZ twins and COR(A_1_, A_2_) = 0.5 for DZ twins. In addition, COR(C_1_, C_2_) = 1 and COR(E_1_, E_2_) = 0, and E(A) = E(C) = E(E) = 0 for all twins. Under the assumption that the genetic and environmental variance components are mutually uncorrelated, the variance of θ can be decomposed as follows:

$${\text{VAR}}\left( \theta \right) = {\text{VAR}}\left( {\text{A}} \right) + {\text{VAR}}\left( {\text{C}} \right) + {\text{VAR}}\left( {\text{E}} \right)$$

where the standardized estimate of VAR(A) is referred to as the heritability, h^2^.

### Homogeneity with respect to measurement occasion

To investigate whether the eight measurement occasions differed in their biometric properties, we relied on estimates of the variance components (A, C, and E) within each occasion. We thus assumed that there are no important differences between the measurement models at each occasion. We fitted the measurement model measurement model and the biometric model simultaneously to the data of each occasion. Note that this was thus a standard ACE model at the level of the latent depression factor (biometric model). On basis of the 99 % highest posterior density regions (HPD) of these variance components, we judged whether the variance components differed importantly over measurement occasion. If not, we concluded that the data collected at the separate occasions were homogenous and could be aggregated.

### Full homogeneity with respect to age

As the age range of our sample is wide (18–70), we also established homogeneity of the data with respect to age. As age is an important moderator in the literature, we wanted a more explicit test on homogeneity than the one discussed above. Here, we followed Nivard et al. (2015), and created age subsamples. We used the following groups: 18–19, 20–21, 22–24, 25–34, and 35–70 years. A major consideration in creating these subsamples was that the sample sizes within each age group need to be large enough to have sufficient power to detect heterogeneity. The resulting number of twin pairs within each age category that were in the analysis (i.e., twin pairs with a full or incomplete data record) is given in Table 2. In the case that the twin members have data in different age categories (due to a twin completing the questionnaire at a different age than his/her co-twin) we omitted their data from the present analysis as it requires independent groups. However, we included their data in the aggregated data analysis.

**Table 2:** The total number of twin pairs within each age group that has been selected for the homogeneity analysis

Age	MZ		DZ			Total	
	Males	Females	Males	Females	Opposite-sex	MZ	DZ
18–19	298 (262)	596 (552)	230 (204)	491 (431)	355 (325)	894 (814)	1076 (960)
20–21	219 (210)	392 (369)	160 (146)	371 (336)	238 (225)	611 (579)	769 (707)
22–24	92 (86)	173 (161)	49 (44)	123 (105)	104 (97)	265 (247)	276 (246)
25–34	77 (70)	248 (229)	46 (38)	108 (94)	114 (103)	325 (299)	268 (235)
35–70	178 (163)	512 (478)	74 (66)	174 (164)	195 (188)	690 (641)	443 (418)
Agg	981 (891)	2165 (2004)	645 (576)	1525 (1349)	1195 (1103)	3146 (2895)	3365 (3028)

The number of twin pairs with a full data record (i.e., with data available for all 13 items in both twins) are in brackets

‘Agg’ denotes the data aggregated over age. If the twin members of the same pair have data in two separate age categories, this pair is omitted from the age grouping to enable multi-group analysis (which requires independent groups). However, this pair is not omitted from the interaction analysis in the aggregated data, leading to data in 6511 pairs for interaction analyses

We tested if some age groups differed importantly from other age groups (e.g., more variance in the phenotypic factor due to heterogeneity). As the age groups are independent (the members of a twin pair are always in the same age group), we could conduct a multi-group analysis and test for homogeneity of the measurement model (i.e., invariant α_i_, τ_i1_, τ_i2_, r_MZ,i_, r_DZ,I_; also referred to as measurement invariance, see Millsap and Yun-Tein 2004) and homogeneity of θ across ages [i.e., invariant MEAN(θ), VAR(θ), and COR(θ_1_,θ_2_)]. First, we tested for invariance of the α_i_, τ_i1_, τ_i2_, r_MZ,i_, and r_DZ,i_ parameters in the five age groups. To this end we fitted the measurement model without the ACE decomposition, but with a correlation between θ_1_ and θ_2_, in the five age groups. We did this separately for the MZ and DZ twins. We started with an unconstrained model (step 0) in which all parameters were free to vary over age groups. Next, step-by-step, we constrained α_i_ (step 1a), r_MZ,i_, and r_DZ,i_ (step 1b), and τ_i1_ and τ_i2_ (step 1c) to be equal across groups. In step 1a we allowed for differences in the variance of θ between the age groups (i.e., we allowed for the possibility that older subjects have a higher/lower variance on the phenotypic depression variable as compared to the younger subjects). To do so, we constrained VAR(θ) = 1 in group 1, and estimated it freely in the remaining groups. In step 1c we allowed for a mean difference in θ between the age groups (i.e., we allowed for the possibility that older subjects have on average higher/lower levels on the phenotypic depression variable as compared to the younger subjects). To do so, we constrained ν = 0 in group 1, and estimated it freely in the remaining groups. If homogeneity with respect to the measurement model is tenable (i.e., step 1c), we could subsequently test for homogeneity of the population model with respect to age. To this end we tested whether the mean and variance of θ (step 2a), and COR(θ_1_,θ_2_) were equal across age groups (step 2b).

### Measurement invariance with respect to gender

As we compared males and females in the interaction models, we analyzed whether homogeneity of the measurement model (measurement invariance) holds for males and females. We did not need to establish homogeneity of θ as we explicitly took possible differences in θ into account in the interaction model. Thus, we assessed whether the parameters α_i_, τ_ic_, r_MZi_, and r_DZi_ were invariant over gender using the procedure from step 1a to 1c as described above. For the MZ subsample this was thus a two group analysis (males–females), and for the DZ subsample this was a three group analysis (male, female, and opposite-sex pairs).

### Testing for interactions in a multi-group model

Recently, within the heteroscedastic ACE methodology (Jinks and Fulker 1970; Molenaar et al. 2012) an approach was presented to enable tests on genotype by environment interactions using the model discussed above (Molenaar and Dolan 2014; Schwabe and Van den Berg 2014). Specifically, retaining the measurement model for θ as discussed above, the biometric model can also be formulated as a conditional model. To this end, we condition θ on A, denoted θ | A. This results in

$$\theta_{\text{pj}} \left| {{\text{ A}}_{\text{pj}} = \nu + {\text{C}}_{\text{pj}} } \right|{\text{A}}_{\text{pj}} + {\text{E}}_{\text{pj}} |{\text{A}}_{\text{pj}}$$

with

$${\text{VAR}}\left( {\theta |{\text{A}}} \right) = {\text{VAR}}\left( {{\text{C}}|{\text{A}}} \right) + {\text{VAR}}\left( {{\text{E}}|{\text{A}}} \right)$$

for the variance decomposition. Now, a genotype by environment interaction is operationalized as an AxC interaction and an A×E interaction:

$${\text{VAR}}\left( {\theta |{\text{A}}} \right) = {\text{VAR}}\left( {{\text{C}}|{\text{A}}} \right) + {\text{VAR}}\left( {{\text{E}}|{\text{A}}} \right) = { \exp }\left( {\gamma_{0} + \gamma_{ 1} {\text{A}}} \right) + { \exp }\left( {\beta_{0} + \beta_{ 1} {\text{A}}} \right)$$

that is, the variance of C and E are made a function of A. Within this function, γ_0_ and β_0_ are the intercept parameters for log[VAR(C)] and log[VAR(E)] respectively which model the size of VAR(C) and VAR(E) at A = 0. In addition, γ_1_ and β_1_ are the interaction parameters, which model the increase or decrease of VAR(C) and VAR(E) across A. The presence of A×C and/or A×E is established by testing whether γ_1_ and/or β_1_ depart from 0. If so, the parameter estimates can be used to infer the direction of the interaction effect. For instance, a β_1_ > 0 denotes that the unique environmental variance is larger for subjects with a higher genetic predisposition, A. In addition, β_1_ < 0 denotes that the unique environmental variance is smaller for subjects with a higher genetic predisposition, A. The same holds for the A×C parameter, γ_1_. This conceptualization of genotype by environment interaction is inspired by Jinks and Fulker (1970), who treated a genotype by environment interaction as an environmental variance that is heteroscedastic across the additive genetic factor. This conceptualization is somewhat different from that of Purcell (2002), who models genotype by environment interactions by making the variance of A a function of a measured moderator (which is not necessarily purely environmental). For a more technical discussion of the biometric model, see Appendix 2.

### Gender effects

As we wanted to allow for gender effects in the aggregated data analysis, an extension of the model by Molenaar and Dolan (2014) was necessary. To account for gender differences in VAR(C|A), VAR(E|A), we used the following parameterization

$$\theta_{\text{pj}} \left| {{\text{ A}}_{\text{pj}} = \nu \times {\text{GENDER}}_{\text{pj}} + {\text{C}}_{\text{pj}} } \right|{\text{A}}_{\text{pj}} + {\text{E}}_{\text{pj}} |{\text{A}}_{\text{pj}}$$

with

$$\begin{aligned} {\text{VAR}}\left( {{\text{C}}|{\text{ A}}} \right) = { \exp }(\gamma_{{0,{\text{overall}}}} + \gamma_{{0,{\text{female}}}} \times {\text{ GENDER }} + \, \gamma_{{ 1,{\text{overall}}}} {\text{A }} + \, \gamma_{{ 1,{\text{female}}}} {\text{A}} \times {\text{GENDER}}) \hfill \\ {\text{VAR}}\left( {{\text{E}}|{\text{ A}}} \right) = { \exp }(\beta_{{0,{\text{overall}}}} + \beta_{{0,{\text{female}}}} \times {\text{ GENDER }} + \, \beta_{{ 1,{\text{overall}}}} {\text{A }} + \, \beta_{{ 1,{\text{female}}}} {\text{A}} \times {\text{GENDER}}) \hfill \\ \end{aligned}$$

where GENDER_pj_ is coded 0 if twin *j* from twin pair *p* is a male and 1 if it is a female. In this way, the new parameters γ_0,female_ and β_0,female_ account for differences in the intercept parameters γ_0,overall_ and β_0,overall_ in the female group as compared to the male group. Similarly, the new parameters γ_1,female_ and β_1,female_ account for differences in the A×C and A×E parameters, γ_1,overall_ and β_1,overall_, in the female group as compared to the male group. Thus, the AxE parameter β_1_ in the male group is equal to β_1,overall_, and the A×E parameter β_1_ in the female group is equal to β_1,overall_ + β_1,female_. The same holds for the AxC parameter. In the model above, the intercept parameter, ν, captures a possible mean difference in θ between males and females.

To account for gender differences in VAR(A) we analogously defined:

$${\text{VAR}}\left( {\text{A}} \right) = { \exp }(\omega_{{0,{\text{overall}}}} + \omega_{{0,{\text{female}}}} \times {\text{GENDER}}).$$

that is, exp(ω_0,overall_) is the variance of A in the male group, and exp(ω_0,overall_ + ω_female_) is the variance of A in the female group.

### Identification and estimation

To identify the model, traditional scale and location constraints were imposed on θ (see Molenaar and Dolan 2014). We identified the scale of θ by fixing α_1_ = 1 for the MZ and DZ twin samples. In single group applications, the location of θ was fixed by imposing ν = 0. As discussed above, in the multi-group model including gender, ν was a free parameter in the female group and fixed to 0 in the male group.

We used a Bayesian approach to model fitting (Eaves and Erkanli 2003). Specifically, we implemented the model in the open-source OpenBUGS software package (Lunn et al. 2009). To this end, we extended the implementation by Molenaar and Dolan (2014) to include the multi-group components as discussed above. The adapted script is available from the website of the first author. Using this script, one can draw samples from the posterior distribution of the parameters using Markov Chain Monte Carlo (MCMC) sampling. From these samples one can determine the parameter means and HPD regions which can be used for statistical inference. The parameters of interest to be estimated were: α_i_, τ_ic_. r_MZi_, r_DZ,i_ β_0,overall_, β_1,overall_, γ_0,overall_, γ_1,overall_, and ω_0,overall_ in the single group analysis. For the multi-group gender analysis we additionally estimated ω_0,female_, β_0,female_, γ_0,female_, β_1,female_, γ_1,female_, and ν. As we used a Bayesian model fitting approach, we specified prior distributions for the parameters. Following Molenaar and Dolan (2014), we used an uniform distribution between −5 and 5 for all parameter except τ_ic_, r_MZi_, and r_DZ,i_. For τ_i1_ we used a uniform distribution between −∞ and τ_i2_; and for τ_i2_ we used an uniform distribution between τ_i2_ and ∞. These priors were meant to ensure that τ_i2_ is larger than τ_i1_ as discussed above. For r_MZi_ and r_DZi_ we specified a uniform prior between 0 and 1 for $\sqrt {r_{{MZ_{i} }} } \;{\text{and }}\;\sqrt {r_{{DZ_{i} }} }$ to prevent sign switching. Note that the missing data in our dataset provide no problem for parameter estimation: In the MCMC procedure these values are considered parameters and are included in the sampling routine. For more technical details concerning the implementation of the model see Molenaar and Dolan (2014).

## Results

### Homogeneity with respect to measurement occasion

Estimates for the contributions of heritability, common and unique environment are given in Table 3. Note that these estimates are based on data from the same that were selected for the interaction analyses as described above. Hence the number of participants in Table 3 is smaller than the total number of twins who took part at each measurement occasion. As can be seen, estimates for heritability tend to be higher than the estimates of 0.4 that are commonly found. Also, there are no differences across measurement occasion in the scaled contributions of the A, C, and E factors to the latent phenotypic depression factor, that is, all 99 % HPD regions overlap. We therefore aggregated the data over the measurement occasions, to obtain a sufficiently large sample size for the heteroscedastic ACE model fitting.

**Table 3:** The proportion of variance explained in the latent depression phenotype by the additive genetic factor (heritability; h^2^), the unique environment (e^2^), and the common environment (c^2^) at each occasion (year of data collection)

Occasion	MZ		DZ		h^2^	e^2^	c^2^
	Twin 1	Twin 2	Twin 1	Twin 2			
1991	290	297	456	450	0.52 (0.37; 0.61)	0.46 (0.38; 0.57)	0.03 (0.01; 0.15)
1995	300	306	426	432	0.62 (0.51; 0.70)	0.37 (0.29; 0.45)	0.02 (0.01; 0.14)
1997	342	335	334	343	0.63 (0.48; 0.71)	0.35 (0.28; 0.45)	0.04 (0.01; 0.21)
2000	472	457	409	420	0.52 (0.35; 0.61)	0.45 (0.38; 0.54)	0.04 (0.01; 0.20)
2002	221	227	178	170	0.51 (0.40; 0.59)	0.48 (0.40; 0.55)	0.03 (0.01; 0.15)
2009	891	896	874	835	0.51 (0.41; 0.58)	0.47 (0.42; 0.53)	0.02 (0.00; 0.11)
2011	144	149	171	172	0.52 (0.37; 0.60)	0.46 (0.40; 0.54)	0.04 (0.01; 0.21)
2013	486	479	517	543	0.50 (0.40; 0.57)	0.49 (0.43; 0.56)	0.02 (0.01; 0.10)

The 99 % Highest Posterior Density regions are in brackets for h^2^, e^2^, and c^2^

### Full homogeneity with respect to age

The RMSEA model fit statistic for the MZ and DZ twin samples is depicted in Table 4 for the different models. As can be seen, all RMSEA values were well below the 0.05 criterion of good model fit (Schermelleh-Engel et al. 2003). Although in both the MZ and DZ twin samples the invariance of τ_i1_ and τ_i2_ was associated with a small deterioration of model fit by 0.002 RSMEA points, there was no obvious source of misfit as indicated by the modification indices (the largest modification index was 11.68 for τ_i1_ of item 4 in the first age group). We therefore concluded that measurement invariance is tenable.

**Table 4:** RMSEA fit statistic for the multi-group models fit to test measurement invariance across the age groups

Step		MZ	DZ
0	Baseline	0.027	0.022
1a	Invariance of α_i_	0.027	0.021
1b	+ Invariance of r_Mzi_ and r_DZi_	0.026	0.020
1c	+ Invariance of τ_ic_	0.028	0.022
2a	No differences in θ	0.028	0.022
2b	No differences in COR(θ_1_,θ_2_)	0.024	0.019

Table 5 contains the estimated means and variances of θ in the different age groups in step 1c. As can be seen, only the mean in the 25–34 age group of the DZ sample was significantly different from zero at a 0.01 significance level. For the variances, only the variance of θ in the 20–21 age group of the MZ sample departed significantly from 1. In addition, restricting the means of θ in all age groups to equal 0 and all variances of θ to be equal to 1 (step 1d) did not deteriorate the model fit, see Table 4. Finally, we tested the latent phenotypic twin correlation, COR(θ_1_, θ_2_) to be equal across age groups (step 1e). As can be seen from Table 4, a model with equal latent phenotypic correlations across age groups improved the RMSEA in both the MZ and DZ twin samples. We therefore concluded that there was no overall age effect detectable. That is, either there is no age effect in the data or the age effect is very small. In both cases we can safely conclude that age did not confound the analysis on the aggregated data as reported below.

**Table 5:** Estimated means and variances of the latent phenotypic factor, θ, in the different age groups

Age	MEAN(θ)		VAR(θ)	
	Estimate	se	Estimate	se
DZ				
18–19	0^a^	–	1^a^	–
20–21	−0.06	0.04	0.92	0.03
22–24	0.14	0.06	0.95	0.04
25–34	0.13	0.06	1.05	0.04
35–70	0.02	0.05	1.01	0.03
MZ				
18–19	0^a^	–	1^a^	–
20–21	0.045	0.053	1.013	0.037
22–24	0.095	0.074	1.118	0.054
25–34	0.217	0.066	1.076	0.046
35–70	−0.116	0.050	1.017	0.037

^a^These parameters are constrained for identification purposes

### Measurement invariance with respect to gender

We started with a baseline model (step 0) in which all measurement model parameters α_i_, τ_i1_, τ_i2_, r_MZ,i_, and r_DZ,I_ were allowed to differ across males and females. Next, we fitted the models from step 1a, 1b, and 1c to the data as discussed above. The results are in Table 6. As can be seen all models fitted well according to the 0.05 criterion. However, in step 1c, the model fit deteriorated notable in both the MZ and DZ twin samples. The modification indices suggested that τ_i1_ of item 3 (‘I cry a lot’) accounts for this misfit (the modification index equaled 112.20 in the male MZ sample). Indeed, as can be seen from the table in step 1c′, freeing this parameter improved the model fit. Results showed that for both the MZ and DZ twins, the threshold parameter τ_i1_ of item 3 was estimated to be much larger for the males as compared to the females indicating that the males tend to use the lower category too often as compared to the females (or similarly, females use the lower category too little as compared to the males). In the final model (step 1c′), the mean difference on θ between males and females (i.e., parameter ν) was estimated to be 0.47 (se 0.04) in the MZ sample and 0.47 (se 0.03) in the DZ sample. In addition, the variance in the female group was estimated to be 1.02 (se 0.03) in the MZ sample and 1.06 (se 0.03) in the DZ sample indicating that there was no variance difference between males and females (the male variance was fixed to 1).

**Table 6:** RMSEA fit statistic for the multi-group models fit to test measurement invariance across gender

Step		MZ	DZ
0	Baseline	0.022	0.018
1a	Invariance of α_i_	0.023	0.020
1b	+ Invariance of r_Mzi_ and r_DZi_	0.022	0.019
1c	+ Invariance of τ_ic_	0.028	0.026
1c’	Free τ_i1_ for *i* = 3	0.024	0.021

This final model without τ_i1_ for item 3 fitted acceptable as compared to the other models. In addition, there was no obvious source of misfit as judged by the modification indices (the largest modification index equaled 12.17 for τ_i1_ of item 4 in the male MZ sample). We concluded that measurement invariance was tenable for all items except item 3. As item 3 was not associated with the same measurement properties for males and females, we omitted this item from the remaining analysis to ensure a meaningful comparison.

### Results of the interaction model

**Fig. 2:**
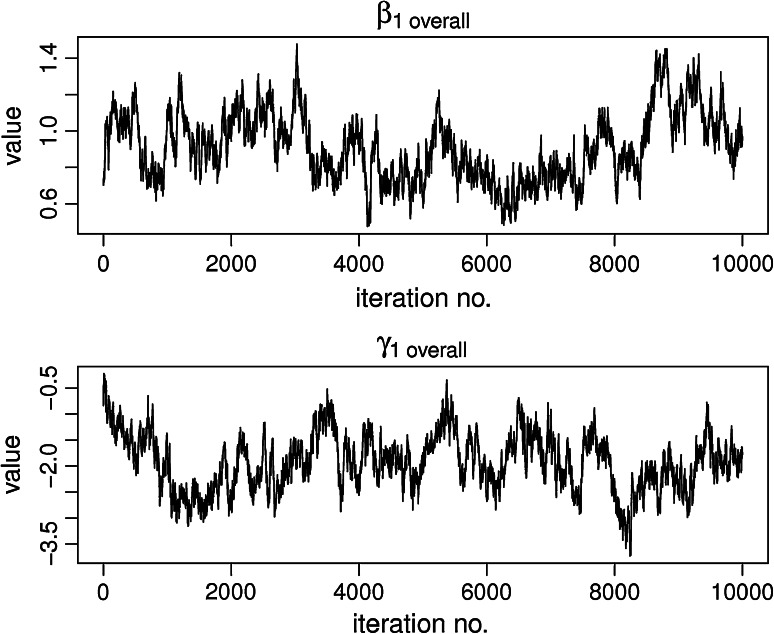
Trace plots of the interaction parameters β_1_ and γ_1_ in the full interaction model in age group 18–19

We drew 20,000 samples from the posterior parameter distribution of which we discarded the first 10,000 as burn-in. From the Gelman and Rubin (1992) diagnostic (based on two chains) and trace plots of the parameters this number of samples appeared to be sufficient to ensure that the chains converged to their stationary distribution. See Fig. 2 for example trace plots of the interaction parameters, β_1,overall_ and γ_1,overall_ of the full interaction model including gender.

We fitted a model without gender differences (i.e., model M1) and a model with gender differences (i.e., model M2, the full gender interaction model). The parameter estimates of the measurement model parameters for model M2 are in Table 7. As can be seen, these correlations were notably smaller in the DZ twin group. This indicates that some item specific genetic and/or shared environmental variance may underlie the scores. By means of the residual correlation, we accounted for this common variance.

**Table 7:** Item parameter estimates (99 % HPD) in the full gender interaction model

Item	α_i_	τ_i1_	τ_i2_	r_DZ,i_	r_MZ,i_
1	1.00^a^	0.96 (0.89; 1.02)	3.10 (3.00; 3.21)	0.12 (0.01; 0.22)	0.22 (0.12; 0.32)
2	1.00 (0.94; 1.07)	1.69 (1.61; 1.78)	3.37 (3.24; 3.52)	0.04 (0.00; 0.16)	0.31 (0.19; 0.42)
3	–	–	–	–	–
4	0.71 (0.66; 0.76)	1.32 (1.25; 1.39)	2.85 (2.74; 2.96)	0.09 (0.01; 0.19)	0.26 (0.16; 0.35)
5	0.52 (0.49; 0.56)	0.09 (0.05; 0.14)	1.47 (1.42; 1.53)	0.12 (0.05; 0.18)	0.31 (0.25; 0.37)
6	0.89 (0.83; 0.96)	1.87 (1.78; 1.97)	3.35 (3.21; 3.50)	0.09 (0.00; 0.22)	0.35 (0.21; 0.48)
7	1.46 (1.36; 1.56)	2.08 (1.96; 2.20)	4.27 (4.06; 4.47)	0.12 (0.00; 0.31)	0.26 (0.12; 0.40)
8	1.00 (0.93; 1.06)	0.35 (0.29; 0.42)	2.68 (2.57; 2.78)	0.04 (0.00; 0.13)	0.26 (0.18; 0.35)
9	1.04 (0.98; 1.11)	1.40 (1.32; 1.49)	3.31 (3.17; 3.44)	0.03 (0.00; 0.13)	0.36 (0.26; 0.46)
10	0.88 (0.83; 0.94)	1.33 (1.26; 1.40)	3.03 (2.92; 3.16)	0.07 (0.00; 0.18)	0.22 (0.11; 0.32)
11	0.85 (0.80; 0.91)	0.57 (0.52; 0.63)	2.44 (2.36; 2.53)	0.14 (0.05; 0.22)	0.32 (0.25; 0.40)
12	1.35 (1.27; 1.45)	1.53 (1.43; 1.64)	4.02 (3.84; 4.22)	0.08 (0.00; 0.21)	0.20 (0.07; 0.33)
13	1.15 (1.09; 1.23)	0.18 (0.11; 0.25)	2.42 (2.31; 2.52)	0.09 (0.00; 0.17)	0.23 (0.14; 0.31)

^a^This parameter has been constrained for identification purposes. In addition, item 3 was omitted from the analysis as it violated measurement invariance across gender

In Table 8, the parameter estimates of the interaction parameters in model M1 and M2 are depicted. As can be seen in model M1 without gender differences, β_1_ departed from 0 and was positive indicating the presence of A×E the variance of E increasing for increasing levels of A. In addition, the γ_1_ parameter did not depart from 0, indicating the absence of A×C in the full sample.

**Table 8:** Parameter estimates (99 % highest posterior density region) of the A×E and A×C parameters in the aggregated data analysis using a model without (M1) and a model with (M2) gender differences in the parameters

	Group	VAR(A)	β_0_	β_1_	γ_0_	γ_1_
M1	–	0.64 (0.53; 0.74)	−0.68 (−0.81; −0.53)	0.34 (0.18; 0.53)	−4.04 (−4.99; −2.43)	−1.38 (−3.05; 1.61)
M2	Males	0.40 (0.25; 0.57)	−0.87 (−1.07; −0.66)	0.91 (0.53; 1.41)	−2.24 (−3.63; −1.45)	−1.93 (−3.26; −0.61)
	Females	0.64 (0.57; 0.73)	−0.65 (−0.78; −0.51)	0.21 (0.003; 0.37)	−5.60 (−7.85; −2.87)	0.54 (−2.90; 3.25)

VAR(A) is calculated as exp(ω_0,overall_) for the males and as exp(ω_0,overall_ + ω_0,female_) for the females. Similar applies to β_0_, β_1_, γ_0_, and γ_1_, see the paragraph on the parametrization of the gender effects

As can be seen in Table 8, when gender differences were taken into account (model M2), a different pattern of results emerged. That is, for both males and females, A×E was present with positive β_1_, but for males, there was evidence for A×C as the HPD region of γ_1_ did not include 0, while for females there was no evidence for A×C. The mean difference between males and females in the latent phenotypic factor, θ, was hardly affected by taking the A×E and A×E interactions into account. That is, ν in the female sample was estimated to be 0.51 (99 % HPD 0.45; 0.59) which was about equal to the estimate reported above in the case of no interactions. It can also be seen from Table 8 that the results from the females follow the results from the entire sample (i.e., M1), while the results from the males are different from the entire sample. We will return to this point in the discussion.

Results in terms of the contributions of heritability, common and unique environment are given in Table 9. Note that these estimates are based on the marginal variance of C and E, as the conditional variance differs across A. The marginal variance for C and E can be calculated using exp(β_0_ + 0.5 × β_1_^2^) and exp(γ_0_ + 0.5 × γ_1_^2^) respectively (see Hessen and Dolan 2009). As can be seen from the Table by taking into account the gender differences in the interactions (M2) the heritability (h^2^) drops from 0.54 in the full sample to 0.35 in the male group. In addition, the contribution of the common environment increases from 0.04 in the full sample to 0.22 in the male group. It should be noted however that the uncertainty in this estimate is relatively large, reflected by the wide 99 % HPD region which runs from 0.09 to 0.37. But at least we can conclude that there is some contribution of the common environment to depression for males.

**Table 9:** The proportion of variance in the latent depression phenotype explained by the additive genetic factor (heritability; h^2^), unique environment (e^2^) and common environment (c^2^) in the full genotype-by-environment interaction model

	Group	a^2^	e^2^	c^2^
M1	–	0.52 (0.43; 0.58)	0.43 (0.37; 0.48)	0.04 (0.01; 0.16)
M2	Males	0.35 (0.21; 0.49)	0.44 (0.33; 0.53)	0.22 (0.09; 0.37)
	Females	0.54 (0.49; 0.59)	0.45 (0.41; 0.50)	0.01 (0.00; 0.06)

Here, c^2^ and e^2^ are the standardized variance of C and E marginally over A

From the results in Table 9 it can be calculated that 33 % of the heritability in males is due to genotype by environment interaction (i.e., 1 − 0.35/0.52; see Molenaar et al. in press). It is clear that this percentage is due to A×C to a large extent, however the exact amount of A×C variance in the male group is difficult to assess. That is, theoretically, the distinction between the effects of A×C and A×E interactions is clear: when not included into the model, regular genetic covariance structure analysis cannot distinguish between an additive genetic factor A and an A×C interaction factor, or between an environmental factor E and an A×E interaction factor (Molenaar et al. 1990). In practical applications however, the parameter estimates for A×C and A×E are correlated which complicate quantification of the exact amount of A×C and A×E variance in the data (see Molenaar et al. 2012).

## Discussion

We studied whether an additive genetic by unique environment interaction (A×E) and/or an additive genetic by common environment interaction (A×C) play any role in adult depression. In a measurement model for categorical item scores, the depression phenotype was operationalized as a latent variable. In a first set of analyses, omitting interaction effects, we found heritability estimates of around 0.5–0.6, which are somewhat larger than those commonly found using an MDD diagnosis or symptom count sum score (i.e., around 0.3–0.4; Sullivan et al. 2000; Nivard et al. 2015). This discrepancy is both of interest and expected, as latent variables always contain less measurement error as compared to observed measures (see Van den Berg et al. 2007).

The present undertaking was aimed at testing the hypothesis that common environmental variance in depression is masked by interaction effects (Duncan et al. 2014). In a single group analysis of the complete sample, we found that the unique environmental variance is larger for individuals with a higher predisposition to develop depression (i.e., higher A factor score). However, we obtained no evidence for A×C interaction in the single group analysis. Next, in a multi-group analysis, we took gender differences into account. We found A×E in both males and females similarly as in the single group analysis, but additionally, there was A×C for the male group. Specifically, in males, common environmental variance is smaller in twins with a higher genetic predisposition to develop depression. The marginal contribution of C increased from 0.04 in the full sample to 0.22 for males by taking gender differences in AxC into account. Although the uncertainty in this estimate is large, it can be concluded that in males, at least some common environment variance is masked by non-linearity due to an A×C interaction.

As mentioned above, the results of the female sample follow the results of the entire sample while the results of the males are different from the results in the entire sample. As interaction effects result in non-normality, the present method detects specific departures from bivariate normality in the latent phenotypic factor θ that are due to A×C (see Molenaar et al. 2012). In the entire sample (collapsing over gender), the non-normality due to A×C in the male sample is masked due to the females that score higher on θ. The distribution of θ in the entire sample is thus approximately normal. In the male sample, the distribution of θ departs from normality resulting in different estimates of the parameters as compared to the entire sample. As there is no A×C interaction in the female sample, the distribution of θ is approximately normal and the results follow the results from the entire sample.

As we argued in this paper, using a measurement model for the item data in testing for genotype by environment interactions may solve the scaling issues commonly encountered in genotype by environment interaction research (Eaves 2006). However, some common limitations of genotype by environment interaction research remain (see Molenaar et al. in press). That is, the presence of a non-linear genotype by environment correlation may conflate the genotype-by-environment interaction. In addition, a genotype-by-environment interaction may spuriously arise if the twin sample is unrepresentative of the population (e.g., the higher phenotypes are underrepresented). Note however that these shortcomings are not unique to the item level approach used here, as they are also problematic in, for instance, the popular genotype by measured environment approach (Purcell 2002).

It would be of interest to model the complete longitudinal dataset including all items at all measurement occasions. That is, the measurement model approach adopted here allows for so-called ‘item linking’ (Kolen and Brennan 2004). In addition, extending the genotype by environment model with a longitudinal component would allow inclusion of the data from all measurement occasions into the analysis, resulting in the largest power possible to detect an interaction effect. However, such an analysis is currently impossible as the required longitudinal genotype by environment models are not yet developed and because of the large sample size, the mathematically complex model, and the tremendous number of missing data in the complete dataset, a full longitudinal item linking approach is numerically intractable.

The present approach provides what may be considered an omnibus test of A×C and A×E, as the interactions are modeled at the level of the latent variables A, C, and E. We consider this an advantage as we do not need to include measured moderators (candidate genes, environmental variables). We emphasize that a failure to detect A×C or A×E using the present method should be interpreted as a result pertaining to A, C, and E. We do not consider the absence of say A×C in females necessarily incompatible with the presence of an interaction detected with a measured moderator, as the power to detect the effect of an interaction with a measured moderator may be greater than the power to detect A×C. The question of which mechanisms underlie gender differences in depression is important. With the present results we hope to have provided a point of departure for further research into the etiology of differences between males and females in the development of depression. Most importantly, we found some empirical evidence for the claim by Duncan et al. (2014) that effects of the common environment underlying depression are masked by non-linear effects. It is therefore advisable to account for these non-linearity when studying the genetic and environmental underpinning of depression.

## Appendix 1

The following 13 items of the anxious depression scale of the ASR were overlapping in all surveys and used in present analysis (translated from Dutch):

I feel lonely (‘Ik voel me eenzaam’),

I feel confused or in a fog (‘Ik voel me in de war of denk wazig’),

I cry a lot (‘Ik huil veel’),

I am afraid I might think or do something bad (‘Ik ben bang dat ik iets slechts zou kunnen doen of denken’),

I feel that I have to be perfect (‘Ik heb het gevoel dat ik perfect moet zijn’),

I feel that no one loves me (‘Ik heb het gevoel dat niemand van mij houdt’),

I feel worthless or inferior (‘Ik voel me waardeloos of minderwaardig’),

I am nervous or tense (‘Ik ben nerveus of gespannen’),

I am too fearful or anxious (‘Ik ben te angstig of bang’),

I feel too guilty (‘Ik voel me erg schuldig’),

I am self-conscious or easily embarrassed (‘Ik schaam me gauw of voel me niet op mijn gemak’),

I am unhappy, sad or depressed (‘Ik ben ongelukkig, verdrietig of depressief’), and

I worry a lot (‘Ik maak me vaak zorgen’).

## Appendix 2

Here we provide the technical details of the model. As discussed, we distinguish between a measurement model for the item scores X_i_, and a biometric model for the latent phenotypic factor, θ. First, as a measurement model we use the graded response model (Samejima, 1969). For the MZ twins, the model is given by:

$$P\left( {X_{{pij}} = c|\theta _{{pj}} } \right) = {\text{ }}\Phi \left( {\frac{{\alpha _{i} \theta _{{pj}} + r_{{MZi}}^{{\frac{1}{2}}} \delta _{{pi}} - \tau _{{ic}} }}{{\sqrt {1 - r_{{MZ_{i} }} } }}} \right) - {\text{ }}\Phi \left( {\frac{{\alpha _{i} \theta _{{pj}} + r_{{MZi}}^{{\frac{1}{2}}} \delta _{{pi}} - \tau _{{i\left( {c + 1} \right)}} }}{{\sqrt {1 - r_{{MZ_{i} }} } }}} \right)\quad {\text{for}}\;{\text{c}} = {\text{~}}0, \ldots ,{\text{Q}}$$

$${\text{with}}\,\tau_{{\text{i}}0} = -\infty\; {\text{and}}\; \tau_{{\text{iC}}= \infty}$$

where Φ(.) is the cumulative normal distribution function, *X*_*pij*_ denotes the score of twin member *j* = *1,2* of twin pair *p* = *1, …, N* on item *i,* and θ_pj_ denotes the latent phenotypic factor. In addition, Q denotes the maximum possible item score (here Q = 2, as we code the data 0, 1, and 2). The parameter *r*_*MZi*_ models the residual correlation between the twin 1 and twin 2 scores on the same item. In the DZ subsample this parameter is replaced by *r*_*DZi*_. As discussed in Molenaar and Dolan (2014), δ_i_ is a standard normally distributed latent variable that is common to X_i1_ and X_i2_ (the scores of the twin 1 and twin 2 members of a twin pair). The loadings of X_i1_ on δ_i_ and X_i2_ on δ_i_ are equal to $r_{MZi}^{{\frac{1}{2}}}$ in the MZ sample and to $r_{DZi}^{{\frac{1}{2}}}$ in the DZ sample. As the residual polychoric variance of X_i_ (i.e., the polychoric variance of X_i_ conditional on θ) is constrained to be equal to 1 - *r*_*MZi*_ for MZ and to 1 - *r*_*DZi*_ for DZ twins (see the equation above), the residual (polychoric) correlation between X_i1_ and X_i2_ (i.e., the polychoric correlation between X_i1_ and X_i2_ conditional on θ_1_ and θ_2_) is equal to *r*_*MZi*_ and *r*_*DZi*_ respectively, see Molenaar and Dolan (2014).

In the biometric model, θ is submitted to the ACE decomposition, that is,

$$\theta_{\text{pj}} = \, \nu + {\text{ A}}_{\text{pj}} + {\text{ C}}_{\text{pj}} + {\text{ E}}_{\text{pj}}$$

where COR(A_1_, A_2_) = 1 for MZ twins and COR(A_1_, A_2_) = 0.5 for DZ twins. In addition, COR(C_1_, C_2_) = 1 and COR(E_1_, E_2_) = 0 for all twins. Under the assumption that the genetic and environmental variance components are mutually uncorrelated, the variance of θ can be decomposed as follows

$${\text{VAR}}\left( \theta \right) \, = {\text{ VAR}}\left( {\text{A}} \right) \, + {\text{ VAR}}\left( {\text{C}} \right) \, + {\text{ VAR}}\left( {\text{E}} \right)$$

As discussed in the main text, genotype by environment interactions are operationalized by conditioning θ on the additive genetic factor A. The conditional variance is given by

$${\text{VAR}}\left( {\theta |{\text{A}}} \right) \, = {\text{ VAR}}\left( {{\text{C}}|{\text{ A}}} \right) \, + {\text{ VAR}}\left( {{\text{E }}|{\text{ A}}} \right)$$

As the variance of C and E are now conditional on A, we can make them a function of A, that is,

$${\text{VAR}}\left( {\theta |{\text{A}}} \right) \, = { \exp }\left( {\gamma_{0} + \, \gamma_{ 1} {\text{A}}} \right) \, + { \exp }\left( {\beta_{0} + \, \beta_{ 1} {\text{A}}} \right)$$

The variance of C and E now depend on the level of A, that is, the variance due to the environment depends on the genotypic factor. Note that because COR(C_1_, C_2_) = 1 by definition, imposing

$${\text{VAR}}\left( {\text{C}} \right) \, = { \exp }\left( {\gamma_{0} + \, \gamma_{ 1} {\text{A}}} \right){\text{ implies that COV}}\left( {{\text{C}}_{ 1} ,{\text{ C}}_{ 2} } \right) \, = { \exp }\left( {\gamma_{0} + \, \gamma_{ 1} \left( {. 5 {\text{A}}_{ 1} + . 5 {\text{A}}_{ 2} } \right)} \right).$$

Thus, in the full model, the vector **A** = [A_1_, A_2_] is distributed as

$$\varvec{A}\sim MVN(\varvec{\mu}_{\varvec{A}} ;\varvec{\varSigma}_{\varvec{A}} )$$

with

$$\varvec{\varSigma}_{\varvec{A}} = \left[ {\begin{array}{*{20}c} {VAR(A)} & {\rho \times VAR(A)} \\ {\rho \times VAR(A)} & {VAR(A)} \\ \end{array} } \right]$$

and ρ = 1 for MZ twins and ρ = 0.5 for DZ twins.

By conditioning on **A**, the conditional distribution of the vector **θ** = [θ_1_, θ_2_] is given by

$$\varvec{\theta}|\varvec{A}\sim MVN(\varvec{\mu}_{\varvec{\theta}} ;\varvec{\varSigma}_{\varvec{\theta}} )$$

with

$$\varvec{\mu}_{\varvec{\theta}} = \left[ {\begin{array}{*{20}c} {A_{1} + \nu } & {A_{2} + \nu } \\ \end{array} } \right]$$

$$\varvec{\varSigma}_{\varvec{\theta}} = \left[ {\begin{array}{*{20}c} {\sigma_{1}^{2} } & {\sigma_{12} } \\ {\sigma_{12} } & {\sigma_{2}^{2} } \\ \end{array} } \right]$$

with

$$\sigma_{1}^{2} = exp\left( {y_{0} + \gamma_{1} A_{1} } \right) + exp\left( {\beta_{0} + \beta_{1} A_{1} } \right)$$

$$\sigma_{2}^{2} = exp\left( {y_{0} + \gamma_{1} A_{2} } \right) + exp\left( {\beta_{0} + \beta_{1} A_{2} } \right)$$

and

$$\sigma_{12} = exp\left( {y_{0} + \gamma_{1} \left[ {\frac{1}{2}A_{1} + \frac{1}{2}A_{2} } \right]} \right)$$.

Now we can condition on θ and specify the distribution of the observed data:

$$X\sim cat\left[ {P\left( {X_{pij} = 0|\theta_{pj} } \right), \ldots ,P\left( {X_{pij} = Q|\theta_{pj} } \right)} \right]$$

where the probabilities in the categorical distribution can be determined using the graded response model above. See Molenaar and Dolan (2014) for a discussion on how these distributions are exactly implemented in OpenBUGS (Lunn et al. 2009).

## Footnotes

1 Molenaar and Dolan (2014) show that even under severe measurement problems (severe floor effects), AxE and AxC tests are relatively unbiased. However, the power to detect an interaction is diminished.
